# Aniline Poisoning

**Published:** 1888-04

**Authors:** Carl Dehio

**Affiliations:** Professor Extraordinary, University of Dorpat


### ABSTRACTS AND EXTRACTS.

### A CASE OF ANILINE POISONING.

BY DR. CARL DEHIO,
Professor Extraordinary, University of Dorpat.

[Berliner Klinische Wochenschrift, No. 1, 1888.
Abstracted and Translated by Professor Lester Curtis.]

A healthy woman, twenty-three years old, having made a good recovery from a perfectly normal delivery, learning that she was about to be discharged from the hospital, took ten grammes of aniline oil at once, with suicidal intent. She then went to bed and apparently went to sleep.

A few hours later the attention of the nurse was directed to her by loud rattling in the throat and groans.

The physician noticed a deep cyanotic coloring of the face and of the extremities, hurried respiration, accelerated pulse, wide pupils, and a penetrating odor of aniline oil from the mouth of the patient.

After instillation of milk, copious vomiting followed. The vomited matter was of a yellow-brown color, and smelled plainly of aniline oil. It was given to Professor Dragendorff for further investigation. About an hour later, light somnolence appeared, which increased rapidly, and toward morning passed into unconsciousness. In this condition the patient was transferred to the medical clinic on the morning of February 13.

There the following conditions were found at nine o'clock in the morning: Powerful, bony frame, abundant sub-cutaneous fat, strong muscles. Patient lies passive on the back, completely unconscious, with closed eyes; reacts neither to calls nor to deep pricks with a needle. Does not swallow when fluid is placed in the mouth. Pupils of medium size, react well toward light. The voluntary muscular activity is fully suspended. The nose cool, hands and feet warm, color of face and skin very pale. The whole surface of the body and the visible mucous membrane is of a peculiar grayish-blue color. No signs of œdema. When the vessels are emptied of blood, by pressure on the skin, the color remains. The color in this respect differs widely from the livid hue of proper cyanosis.

The breathing, 25 in a minute, is accelerated, irregular, and unequal, the inspirations for the most part uncommonly deep, the expiration protracted and occasionally attended with groans. The pause between inspiration and expiration is lacking; voice during groaning clear, no cough. The boundaries of the lungs normal. On the posterior dependent portions some moist medium bubbling, soft crepitant râles and sibilant râles, every where vesicular breathing, nowhere muffled.

Pulse, 132, with low wave, radial artery imperfectly filled and tension low, apex-beat in the fourth intercostal space, within the line of the nipple, not enlarged nor strengthened. No epigastric pulse, heart sounds clear and plain, spleen and liver show nothing abnormal. Abdomen tympanitic throughout; tongue moist, mouth and fauces unchanged except from the blue coloration of the mucous membrane. On the buttocks a few small venous ektasia.

The stomach was immediately washed out, but brought to light no trace of stomach contents (patient had vomited a few hours before). Temperature of the body not raised (36.9°) (98.4°Fahr.)

The diagnosis was made perfectly certain by Professor Dragendorff, who obtained with the greatest clearness, in the vomited matter, the reaction of aniline as well as that of toluiden.

The clinical history is as follows:

The first twenty-four hours the condition of the patient remained nearly unchanged. Every three hours the patient was put in a warm full bath of 30° R. (99.5° Fahr.), and while in the bath, ice water was poured over head, breast, and back, but with no results.

Turpentine inhalations, recommended by Kiljanski in aniline poisoning, were used without producing any change in the condition. The pulse was small and easily compressible, and varied from 124 to 136 beats; sphygmographically, it showed a plain lowering of the pulse-wave during inspiration. The temperature of the body sank during the course of the day to 35.7° C. (96.2° Fahr.)

The amount of urine drawn with the catheter about 2 p. m., about fourteen hours after the poisoning, amounted to 585 ccm. (1 pint), had a specific gravity of about 1,019, was somewhat turbid, of a deep yellow color, with a strong acid reaction, and contained neither albumen nor sugar. Biliary coloring matter could not be shown in the urine. With the microscope leucocytes and some bladder epithelium were found.

The investigation of this portion of the urine, undertaken by Professor Dragendorff, gave aniline and toluidin (especially paratoluidin), as well as the presence of derivatives from both of these substances which had probably been separated by the kidneys in the form of complex sulphonic acids (gepaarten Schwefelsäuren).

About 10 o'clock in the evening, about eighty ccm. (two ounces) of blood were taken from an opening in a vein. This was very dark and brownish, and was fluid after ten minutes. The serum, which by the next morning had separated from the coagulum, was of an intense yellowish red. The examination of this for an accidental biliary coloring matter was unfortunately neglected by me. Professor Dragendorff has shown in the blood aniline and toluidin, and the same derivatives from these substances as in the urine.

During the night of February 13–14 the breathing was quieter, and the complete unconsciousness passed into a state in which the patient still lay in a deep stupor, but noticed, though imperfectly, strong irritations. At least, after deep pricks with the needle, slight reflex twitchings occurred and weak motions of warding off.

On the morning of February 14 (about thirty hours after the poisoning) the temperature was 37° (98.6° Fahr.), the pulse was 112, the wave very low; the radial artery had the same weak tension and fullness as the day before. The respiration was still accelerated (36 a minute), but more uniform, and no longer accompanied by groaning. The surface of the body moist, the forehead and face covered with profuse sweat; consciousness had not yet fully returned.

Toward noon, the patient began to swallow when fluids were put into the mouth. After an enæma, followed a tolerably copious brownish-yellow stool.

At six o'clock in the evening the patient lay in a copious perspiration, which covered the whole body and collected in thick drops on the face. She opened her eyes and looked before her with an apathetic expression; did not speak; did not move spontaneously, and responded to needle pricks by sluggish shrinking and groaning sighs. Pulse 104, wave very small, scarcely perceptible; heart sounds faint but clear; respiration 32, superficial; some cough, everywhere vesicular breathing; crepitant râles not noticeable; extremities warm, temperature 33.7° (92.° Fahr.)

Ordered a tablespoonful of brandy punch every hour.

On the morning of the 15th of February, three days after the poisoning, intelligence had fully returned. Patient answers all questions sluggishly, but with perfect clearness; complains of headache and sense of oppression in the left of the abdomen. Voluntary motions are possible; but the general weakness of the body is so great that she can not turn herself on her side without help, or hold her arm in the air unsupported. Pricks with the needle are perceived accurately everywhere; pupils torelably wide, react well to light. The pulse, 108, is fuller and stronger than last evening. Breathing free, 26.

The jaundiced coloration of the skin and conjunctiva has increased. The liver not demonstrably increased, not painful on pressure. Spleen the same. The blue coloration of the skin has become less; but in spite of the contemporaneous jaundice it is plainly noticeable under the breasts, on the nates, and in the inguinal folds. Temperature, 37.5° (99.5° Fahr.)

Ordered brandy, buillon, milk.

The urine drawn with the catheter at 11 a. m. amounted to 485 ccm. (sp. grav. 1,020), was dark yellow red, clear, free from albumen, and gives a plain reaction for biliary coloring matter.

February 16, morning (fourth day of illness). Patient is indifferent to everything that goes on around her, headache less, no thirst, no appetite, an abundant secretion from the vagina, jaundice and general condition unchanged, temperature 37.2° (98.6° Fahr.), pulse 90, respiration 30.

Evening, temperature 37.2° (99° Fahr.), pulse 88, respiration 28. Patient complains of severe spontaneous pain, and of pain, on pressure, in the epigastrium. No nausea, vomiting, or other gastric symptoms, with exception of loss of appetite. Patient has had no evacuation of the bowels or of the urine for twenty-four hours, and was therefore catheterized. The urine thus obtained was 1,325 ccm. (2 4-10 pints), sp. grav. 1,020, is brown red, somewhat turbid, acid; the filtered urine does not become cloudy on boiling, and gives a plain reaction for biliary coloring matter.

February 17, morning (fifth day of illness). Temperature, 36.8° (98.3°Fahr.), pulse 88, respiration 24. Patient is perfectly sensible, complains of slight headache. Pain in epigastrium on pressure is less, jaundice less intense, surface of the body lemon-yellow color, pulse small and weak, respiration perfectly free and quiet.

Evening. Patient has again passed no urine for twenty-four hours, although the bladder is distended almost to the navel. She has raised the suspicion, by her capricious, hysterical manner, that the retention of urine is simulated. About 1,465 ccm. (2.6 pints) of urine were drawn by the catheter at one time, which was of the same character as the day before; temperature 37.6° (99.7°Fahr.), pulse 80. A hypodermic injection of morphine was given in the night on account of a sudden cardialgia.

February 18, morning (sixth day). Temperature 36.7° (98.3°Fahr.), pulse 80. The patient is weak and without appetite, complains of moderate abdominal pain. Objectively, everything is unchanged, the retention of urine continues, the jaundice is fainter than the day before, the blue coloration no longer noticeable, the urine, drawn at evening with the catheter (sp. grav. 1,018), amounts to 825 ccm. (1.5 pints). It is very turbid, brownish-red, acid, and on standing, deposits an abundant muco-purulent sediment. Filtered clear and boiled, it shows a scanty red cloud which does not diminish by saturation with acetic acid (hæmoglobin?) Notwithstanding the dark color of the filtered urine, Gmelin's test gives no plain reaction for biliary coloring matter. Temperature 37.4° (99.3°Fahr.), pulse 84.

February 19, morning (seventh day). Patient slept well during the night; temperature 37.5° (99.5°Fahr.),pulse 108, respiration 26, appetite returns. Tenderness, on pressure, over the epigastrium unchanged, liver not demonstrably enlarged, jaundiced color of the body still present. Patient is still so weak that she can not stand, but she can hold herself upright in bed for a short time. The patient passes no urine voluntarily, and complains of no feeling of oppression, although the bladder is distended almost to the navel. With the catheter 1,700 ccm. (3 pints) (of 1,014 sp. grav.) of urine were drawn. This has to-day a quite striking appearance; it is of a black-brown color, almost perfectly opaque, of an acid reaction, and on standing, precipitated an abundant red-brown sediment. When filtered, the urine is of crystal clearness, of a Burgundy-red color and, with the spectroscope, shows in the plainest manner the absorption bands of oxyhæmoglobin. By boiling, there collects a floating, coagulated, brown mass. When this is filtered out, the urine appears a pale yellow. Heated with a solution of potash, a red-colored, rather scanty, sediment is deposited from the filtered urine, without a perfect decolorization of the urine resulting (Heller's blood test). Biliary coloring-matters are no longer to be demonstrated with certainty. The urine sediment consists of very numerous round cells, which in part contain a large plain nucleus and have taken up a great quantity of fine brown granules. Some of these cells contain vacuoles. Further, a quantity of cylindrical structures are to be noticed, of the size and form of medium-sized tubecasts, which consist of dark-brown granular masses; also a few hyaline casts, which are covered with thickly granular brown masses. A sample of blood was obtained from a small venesection; it flowed readily, and the following day showed a deep Burgundy-red serum in which was seen oxy-hæmoglobin spectrum.

A sample of blood from the tip of the finger shows, under the microscope, a defective formation of rouleaux, some half decolorized blood-corpuscles, and a good many remains of red corpuscles which have become quite colorless (so-called shadows); dismembered poikilocythen, among which the cup-shaped, bulged-out forms prevail; white blood-corpuscles, relatively quite numerous, and a quantity of small colorless nuclei, and heaps of granules. Several counts of the red blood-corpuscles gave an average of 2,700,000 corpuscles in a cubic millimetre.

February 20, morning (eighth day). Patient has slept tolerably well; temperature 37.5° (99.5°Fahr.), pulse 95, appetite good, body weakness still very great, jaundice diminishes constantly. Evening, temperature 36.7° (97.°Fahr.), pulse 104, respiration 20; 1,500 ccm. (2.7 pints) of urine were spontaneously voided. The color is dark red, a little brighter than yesterday. In other respects the urine shows exactly the same character as the day before.

February 21, morning (ninth day). Patient slept badly, complains of headache, temperature 36.7° (91.°Fahr.), pulse 96. There is now only a slight trace of jaundice, the general condition of strength improves, has appetite. The urine is now voided spontaneously, the daily quantity amounting to 1,900 ccm. (3.4 pints).

The urine is acid, turbid, and of a red color, but brighter than yesterday, and allows only a slight red color of the precipitate to be seen with Heller's blood-test. Spectroscopically, the hæmoglobin bands are less distinctly to be noticed; biliary color reactions are no longer to be obtained; the abundant sediment consists of leucocytes and manifold nuclei, which contain within them a number of colorless, strongly refracting granules and drops and only a few brown granules.

February 22, morning (tenth day). Temperature 36.4° (97.5°Fahr.), pulse 110. Evening, temperature 37.3 (99.°Fahr.), pulse 132. The urine is of a yellow color, turbid. Hæmoglobin is no longer to be demonstrated. The daily quantity amounts to 1,550 ccm. Brown granules are no longer to be found in the cells of the copious purulent sediment.

February 23, morning (eleventh day). Temperature 36.6° (97.9°Fahr.) Evening, temperature 37.3° (99.°Fahr.), pulse 105. Jaundice has entirely disappeared, the great degree of wax-like paleness of the skin and mucous membrane now shows very plainly.

The quantity of fat has decidedly diminished in the past week, the muscular apparatus is weak and relaxed, the appetite moderate, no stomach symptoms. Objective changes in the abdominal organs can not be demonstrated. Belly soft, not springy, liver and spleen not demonstrably changed, patient complains of severe pain in the whole abdomen, and has been constipated for a few days. The blood drawn from the forefinger by a needle-prick is microscopically very pale and watery, and shows an average of 1,400,000 red corpuscles in a cubic millimetre. Quantity of urine 1,250 ccm., with the same character as yesterday. A soft brown stool followed a dose of castor oil.

February 24, morning. Temperature 36.8° (298.°Fahr.), pulse 90. Evening, temperature 37.2° (99.° Fahr.), pulse 132.

Quantity of urine 1,400 ccm. (2½ pints), character unchanged; sleeplessness. After 4.0 paraldehyde, sleep followed for two hours.

February 25. Condition unchanged; quantity of urine 1,450.

Evening, paraldehyde 6.0, followed by some hours' sleep.

February 26, morning (fourteenth day).

Temperature 36.9° (98.4° Fahr.), pulse 99. The patient is very languid and without strength, and complains continually of drawing pains in the body and the extremities, and of occasional numbness in the latter. Emaciation has made rapid progress and the anæmia has reached a very high degree. The skin is pale as marble, the lips quite colorless.

The urine is of a deep yellow color, is feebly acid, and, on standing, becomes very soon alkaline. It deposits a thick muco-purulent sediment, and contains in the purulent mixture a correspondingly small quantity of albumen. The sediment consists of leucocytes, flat epithelium, and disintegrated hyaline tube-casts with a few casts beset with epithelium.

From February 27 to the 1st of March, the condition of the patient gradually improved; the strength began to increase, but the vague pains remained, and the urine also contained a muco-purulent sediment. Tube-casts were no longer to be found.

March 2 (eighteenth day). A moderate but unmistakable diminution of the liver dullness can be demonstrated. Its lower border is two finger-breadths higher than normal, and reaches on the left only to the median line, so that the lower edge of the heart-dullness borders, to a great extent, directly on the tympanitic resonance of the stomach. The examination of the urine for leucin and tyrosin gives a negative result. Two counts gave respectively 3,000,000 and 3,400,000 blood-corpuscles in a cubic millimetre of blood.

March 3. The day's quantity of urine amounts to 1,650 ccm., and in this are contained :

Urea...........( 1.06 p. ct.)=18.02 gr.

Uric acid.......(0.0156 p. ct.)= 0.27 gr.

Chlorine........( 0.072 p. ct.) 1.97 g. na. cl.

Sulphur ($SO_3$)..( 0.04 p. ct.)=68. g.

Phosphorus ($P_2O_5$) ( 0.08 p. ct.)=1.36 g.

While in general the secretion of the ordinary constituents of the urine had returned to about half the normal, which in the anæmic patient lying in bed is not to be wondered at, the amount of chlorine separated in the twenty-four hours is about one-tenth the normal daily secretion. An analysis of the urine from March 17 to 22, gave quite normal results. On the 7th of April, patient was discharged.

The author's discussion of this history, though occupying some space, must be given very briefly.

Following the first vomiting, which was undoubtedly due to gastric irritation, were the symptoms which were, without doubt, due to the action of the poison contained in the blood on the nervous system, such as the stupor, the loss of the reflexes, and power of motion, the change in breathing, pulse-rate, depression of temperature, the sweating, etc.

The bluish hue of the skin appears to be due to a coloring matter formed from the breaking up of the aniline oil. Dragendorff is also of this opinion. Similar substances were found in the urine, but disappeared after the fifth day, when the aniline oil seems to have been pretty completely eliminated.

The poison appears to have had a destructive action on the blood. Twenty-one hours after the poisoning the urine contained biliary coloring-matters; the jaundice grew in intensity up to the fifth day, when the biliary coloring-matter disappeared from the urine, and the jaundice diminished. The opinion of the author is that this color comes from disintegrated blood-corpuscles. If it had been due to absorption of bile there would have been more biliary acids in the urine.

On the sixth day the urine contained a great quantity of hæmoglobin which, of course, indicated a still greater degree of destruction of the corpuscles. How great this destruction was is indicated in several ways, especially by the count of the red corpuscles, which, instead of the normal 5,000,000 to 5,500,000 in a cubic millimetre fell on the 11th day to 1,400,000.

From the ceasing of the hæmoglobinuria dates the beginning of the convalesence of the patient.

"At any rate, the present clinical case shows with a sharpness which can not be excelled by an experiment on an animal, that aniline oil belongs to that group of poisons which can cause both jaundice and hæmoglobinuria in the same individual; a fact which before this time was not known."

